# Supplementary material for: Drug and disease signature integration identifies synergistic combinations in glioblastoma
Source: Nat Commun. 2018 Dec 14;9:5315. doi: 10.1038/s41467-018-07659-z (PMC6294341; doi:10.1038/s41467-018-07659-z)
Supplement: Supplementary file 1 — Supplementary Information [file 41467_2018_7659_MOESM1_ESM.pdf]

SUPPLEMENTARY METHODS

Generating L1000 Signatures for Glioblastoma

The LINCS L1000 March 2017 dataset contains transcriptional profiles of 30 cell lines treated with 1768 small molecules. However, even though most cancer types are represented by multiple cell lines, the dataset did not contain any Glioblastoma (GBM) cell lines. For this, we submitted GBM samples to be processed by the L1000 platform. Two GBM Patient Derived Xenograft (PDX) cell lines and four GBM stem-like cell lines were treated with a selection of compounds and had their RNA sent to be processed via the L1000 assay (Data available at [http://dx.doi.org/10.17632/yz8m28gj6r.1]).

In order to quantify the level of transcriptional activity induced by each perturbagen, we calculated the Broad TAS (Transcriptional Activity Score):

$$TAS = \sqrt{SS \times \max(CC, 0)\,/978}$$

Supplementary Equation 1

where SS is the Broad’s Signature Strength score (number of differentially expressed genes in a L1000 signature) and CC is the BROAD’s Replicate Correlation score (a measure of technical replicate reproducibility) (Supplementary Figure 1c). Furthermore, for each compound, we calculated Transcriptional Consensus Signatures to identify genes that were consistently expressed in GBM cells. In Supplementary Table 1, a list of the consistently over and underexpressed genes for the five conditions treated in GBM cells are shown.

Synergy Screen in Glioblastoma PDX Cell Lines

The synergy screens for JQ1 with GSK-1070916, JQ1 with SR1277, gemcitabine with mitoxantrone, and gemcitabine with imatinib consisted of a minimum of three technical replicates normalized to a positive (Velcade) and negative (DMSO) control to calculate the percentage of cell death (Supplementary Data 1). As an example of the output from the SynergySeq application, we have shown the results for the combinations of FDA-approved compounds with gemcitabine (Supplementary Data 2). The Orthogonality and Discordance scores from the output file were normalized to the subset of 84 compounds and the Orthogonality Score (OS) was computed. This analysis provided a strong correlation between the discordance and the measured percentage of cell death (Figure 7b-d).

Comparison of TCSs with CLUE Connectivity Scores

We wanted to evaluate the biological relevance of the Transcriptional Consensus Signatures by comparing it with BROAD's cell-summarized connectivity score[1]. This score can be used as a perturbagen-specific metric that summarizes the connectivity scores of individual cell lines and ranges between -100 to 100. A higher score indicates a higher transcriptional-response similarity between 2 compounds. We downloaded the summarized connectivity scores from [https://clue.io/] for 2837 small molecule IDs (BRD IDs) and created a highly-curated list of the Mechanisms of Action associated with those BRD IDs using annotations found in CLUE, DRH[2], CTRP [3], and CHEMBL[4]. Our list of 724 unique MOAs covered 2549 out of the 2837 BRDs from CLUE (Supplementary Data 3).

We then obtained the corresponding Phase 1 L1000 Level 4 data from GEO [https://www.ncbi.nlm.nih.gov/geo/query/acc.cgi?acc=GSE92742] and created TCS for the above compounds (method in main text).

To make a more robust small molecule reference list, we included MOA categories that included at least 3 BRD IDs and TCS for BRD ID that contained at least 40 genes per each TCS. Similar to the Phase 2 TCSs, many compounds have a low number of genes that are deregulated consistently across different experimental conditions (Supplementary Figure 7). The 40 gene threshold was chosen to maximize the number of compounds that we will use further in the analysis while keeping a relative high number of genes in each TCSs and we find 917 BRD IDs that are associated with a rich MOA annotation list and have a unique TCS.

We then proceeded to evaluate how well BROAD's connectivity score and the Concordance Ratio (Equation 2, main text) could measure the transcriptional correlation of compounds belonging in the same MOA category. In addition to concordance ratio, we evaluated the Spearman correlation of the TCSs.

For every MOA category we calculated the average connectivity score, concordance ratio, and spearman correlation between compounds belonging to the same category and compared it to a calculated null distribution of 10000 permutation of the genes for each small molecule signature. Using this empirical null distribution, we calculated the z-score that represented the distance of each MOA from its respective null distribution.

As shown in Supplementary Figure 7, the TCS have comparable results to the Broad's connectivity score, with the added advantage that for each compound apart from a

similarity score, we also obtain a list of biologically relevant up and down-regulated genes that can provide the same level of information as the single CLUE similarity metric.

Individual TCSs Can Predict Sensitivity in Glioblastoma Cells

To evaluate whether the TCS could be used to predict the response of Glioblastoma cells to the respective drug, we used a publicly available screening of 446 FDA approved compounds using Glioblastoma cell lines[5]. Out of the 446 compounds, 194 compounds were used in the LINCS L1000 dataset and for each drug, we calculated its respective TCS. As previously reported, a large portion of the drugs had a weak transcriptional response and contained a low number of genes in their TCS (Supplementary Figure 8). We then focused on compounds that had at least 40 genes in their TCS. Compounds that had an anti-proliferative effect in Glioblastoma cells, had a significantly higher Discordance Ratio score, Supplementary Figure 8 (Wilcoxon rank sum test, p-value = 0.0001862). Importantly, was the observation that many of the compounds with a high Discordance Ratio but were not labeled as active in Jing 2014, have been shown to have an antiproliferative effect in other Glioblastoma model systems (rimcazole: Discordance Score = 4, olanzapine: Discordance Score = 2)[6].

Comparing Large Combination Screening Datasets

We next evaluated our pipeline by analyzing two combination oncology screens (the NCI ALMANAC Dataset [7] and the Oneil et al Dataset [8]). The NCI ALMANAC Dataset reports a systematic screen of over 5000 pair-wise combinations using 104 FDA approved drugs in 60 cell lines (NCI60). In the Oneil Dataset, 38 compounds were used in 583 pairwise

combinations to treat 39 cancer cell lines. To measure the combination benefit, we used the ComboScore for the NCI ALMANAC Dataset and the Lowe Additivity Score for the Oneil Dataset. ComboScores are available through the NCI ALMANAC data portal [https://dtp.cancer.gov/ncialmanac/] and the Lowe Additivity Score was obtained from a previous publication[9]. As previously reported in small scale combination screenings [6], the distribution of synergy scores follows a normal distribution, centering near 0 (Supplementary Figure 9). To adjust for the use of different synergy metrics between the two datasets, we calculated the z-scores from the two metrics.

Since the reproducibility across different single-dose datasets has been a widely acknowledged issue[10-15] , we proceeded with evaluating the consistency between the NCI ALMANAC and the Oneil dataset. As we could not detect any observable correlation between the two datasets, we proceeded with using the larger NCI ALMANAC dataset (Supplementary Figure 9c-f).

Finally, we wanted to validate the synergistic potential of drug combinations that have a high Discordance Ratio (Equation 3, main text), by using the NCI ALMANAC Dataset. We filtered for compounds that had a TCS of at least 40 genes, which yielded a list of 657 LINCS compounds. We also filtered clinically relevant drugs that showed at least 10% decrease in Cell Growth (PercentGrowth <90) when used in single-agent treatments of the cell lines. We identified 19 compounds that were used in pair-wise combinations in 33 cancer cell lines in the NCI ALMANAC Dataset. Although all combinations with a ComboScore above 0 are technically synergistic, we followed a more conservative approach, similar to a previous method [9], where we label as synergistic, the combinations

with the top-ranked ComboScore values (top 5%) and as Negative, all the antagonistic, additive and low synergistic drug combinations.

In Supplementary Figure 10 we can see that drug combinations labeled as synergistic show a significantly higher Discordance Ratio (Wilcoxon rank sum test p-value = 8.808e-11), compared to combinations that are labeled as negative. Moreover, we can see a cancer type-dependent distribution of the Discordance Ratio, where most cancer types, synergistic combinations show a higher Discordance Ratio than negative combinations. We also assessed the effect of the z-score threshold when discriminating between synergistic and negative combinations, by evaluating the median of the corresponding groups using different thresholds, indicating that regardless of the z-score threshold, Synergistic combinations have a significantly higher Discordance Score than the Negative combinations. Finally, we calculated the distribution of the difference in medians between the two categories after 200,000 permutations of the synergy and negative labels (permutation p-value= 4.9999e-06) and we plotted the corresponding histogram. The dotted line indicates the difference in the medians of the correct labeling.

Consensus Clustering on TCS

Similar to Figure 4, we utilized the LINCS L1000 March 2017 Dataset and calculated the Pearson correlation between the TCSs and employed the widely-used consensus clustering algorithm[16] to identify robust and stable clusters of compounds. For this, all compounds that had at least 10 genes in their TCS (1408 compounds) and performed consensus clustering using the ConsensusCluster Plus R package (number of subsamples: 1000, clustering algorithm: hierarchical, Distance Euclidean, proportion to

sample: 0.8). As we can see in Supplementary Figure 2a, compounds with similar MOAs cluster together. Of note is cluster 14, where both GSK-1070916 and alisertib are robustly clustered together and cluster 32, where all the BROAD Bromodomain inhibitors are clustered together with Glioblastoma JQ1 TCS.

The list of compounds with their respective cluster number and the MOAs are available at Supplementary Data 4.

Transcriptional Consensus Signatures of Known Aurora Kinase Inhibitors

To evaluate the transcriptional-response similarity between known Aurora Kinase Inhibitors, we calculated the Concordance ratio (Equation 3, main text) among each Aurora Kinase inhibitor and all compounds in the LINCS L1000 March 2017 dataset. The compounds with the highest Concordance Ratio are shown in Figure 4b. If a compound was another Aurora kinase inhibitor, it was labeled with yellow and the GSK-1070916 compound was labeled with red. The complete list of Concordance Ratios for each Aurora kinase is available at Supplementary Data 5. Moreover, the significance of the similarity was evaluated by permuting 10,000 each TCS and calculating a permutation p-value for each Concordance Ratio.

SUPPLEMENTARY FIGURES

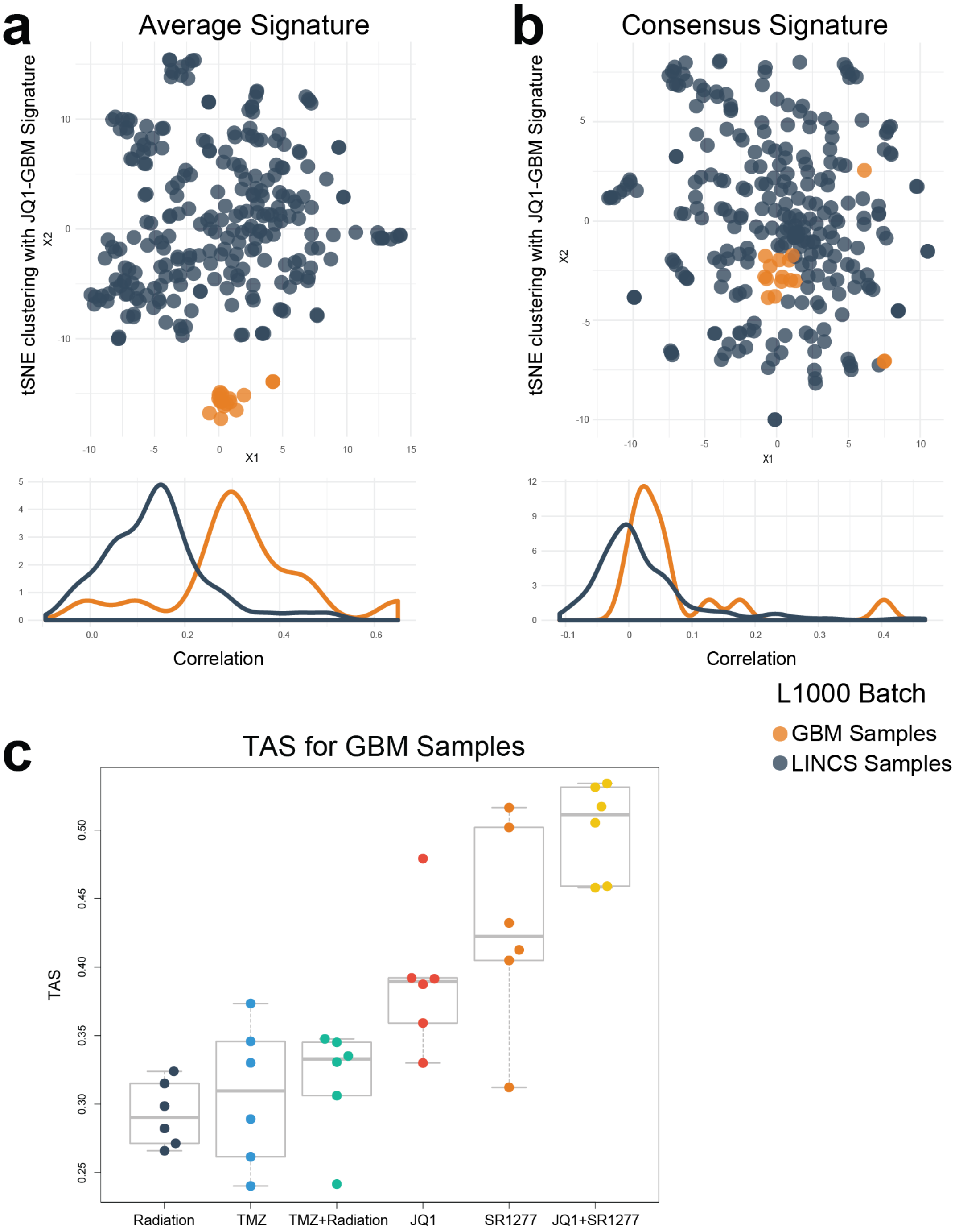

Supplementary Figure 1. Reduced batch effect using consensus signature compared to average signature. tSNE plot (top) and distribution of correlations (bottom) of the correlation of the GBM samples compared to all LINCS samples using **a.** the average signature method and **b.** the consensus signature method. **c.** Boxplots of the TAS (Transcriptional Activity Score) for the GBM samples. Box and whisker plots show the median (horizontal line), the 25th and 75th percentiles (lower and upper bounds of box), extremes (whiskers), and outliers (●).

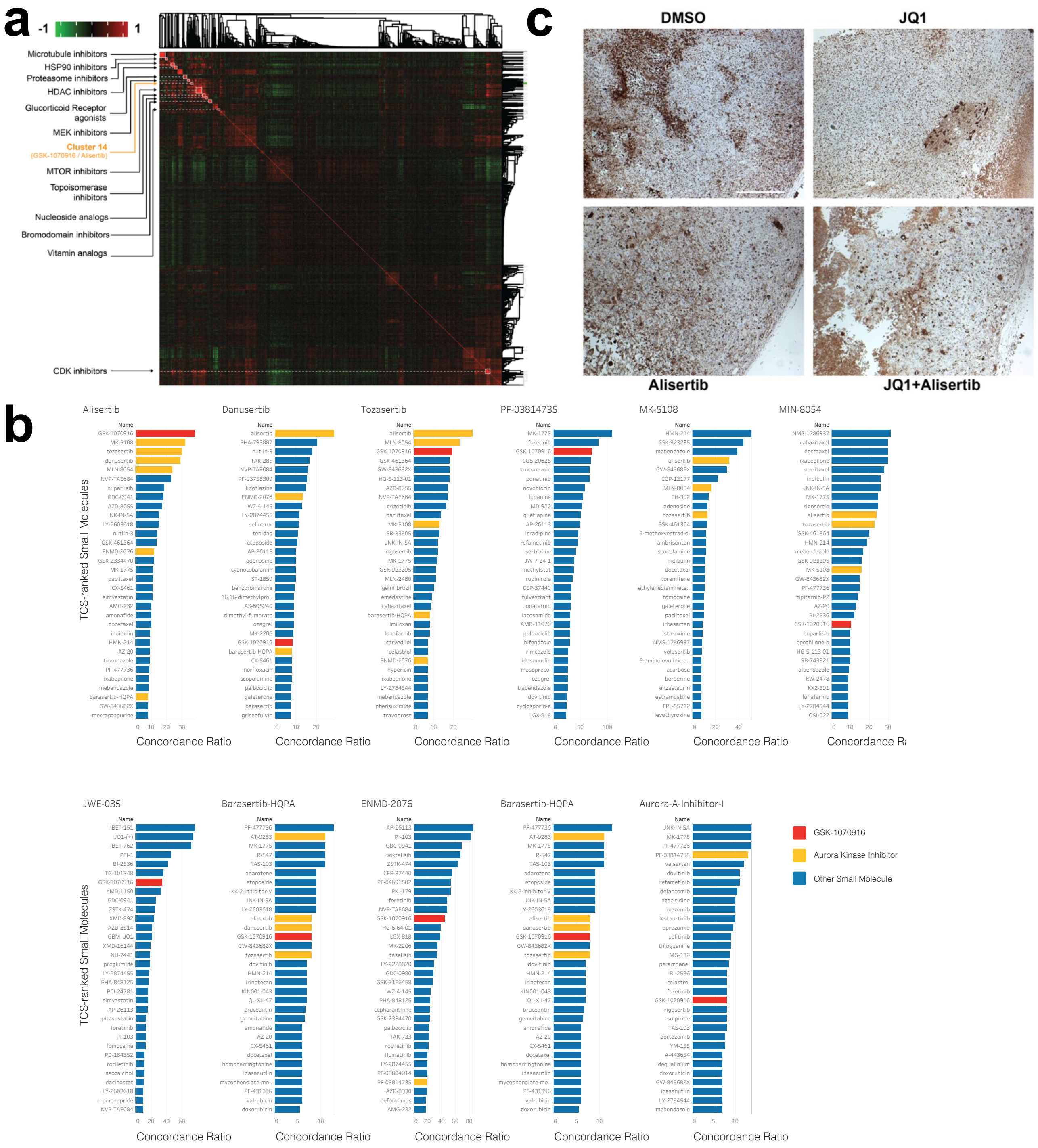

Supplementary Figure 2. Alisertib is an aurora kinase inhibitor that synergizes with JQ1. a. Consensus clustering[16] of TCS for LINCS L1000 compounds. Note that GSK-107096 and alisertib are in the same cluster, cluster 14. **b.** Ranking of TCS similarity for all aurora kinase inhibitors. As seen in (a), the TCS for Alisertib is similar to GSK-107096. **c.** JQ1+Alisertib combination reduces GBM tumor cell proliferation *in vivo,* related to Figure 6. Tumors were isolated from mice treated in Figure 6 and processed for Ki67 staining, which marks proliferating cells. Note that the JQ1+Alisertib combination had less Ki67 staining than other treatments. Scale bar=500μM**.** Ki67 staining was performed at the University of Miami Sylvester Comprehensive Cancer Center core using the Bond Max Immunostainer.

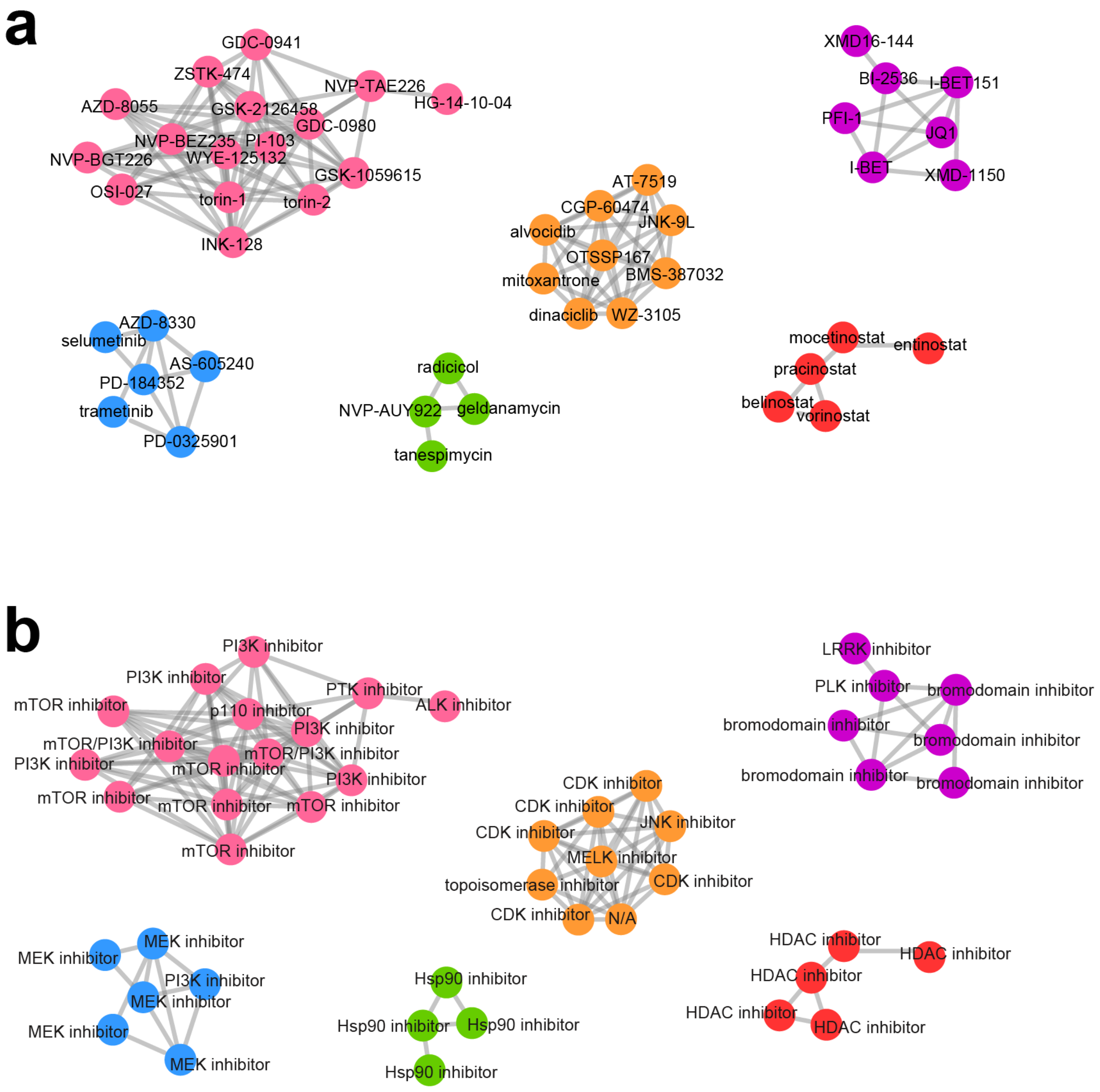

Supplementary Figure 3. A scaled view of each inhibitor sub-network. Nodes are labeled by **a.** compound name and **b.** mechanism of action.

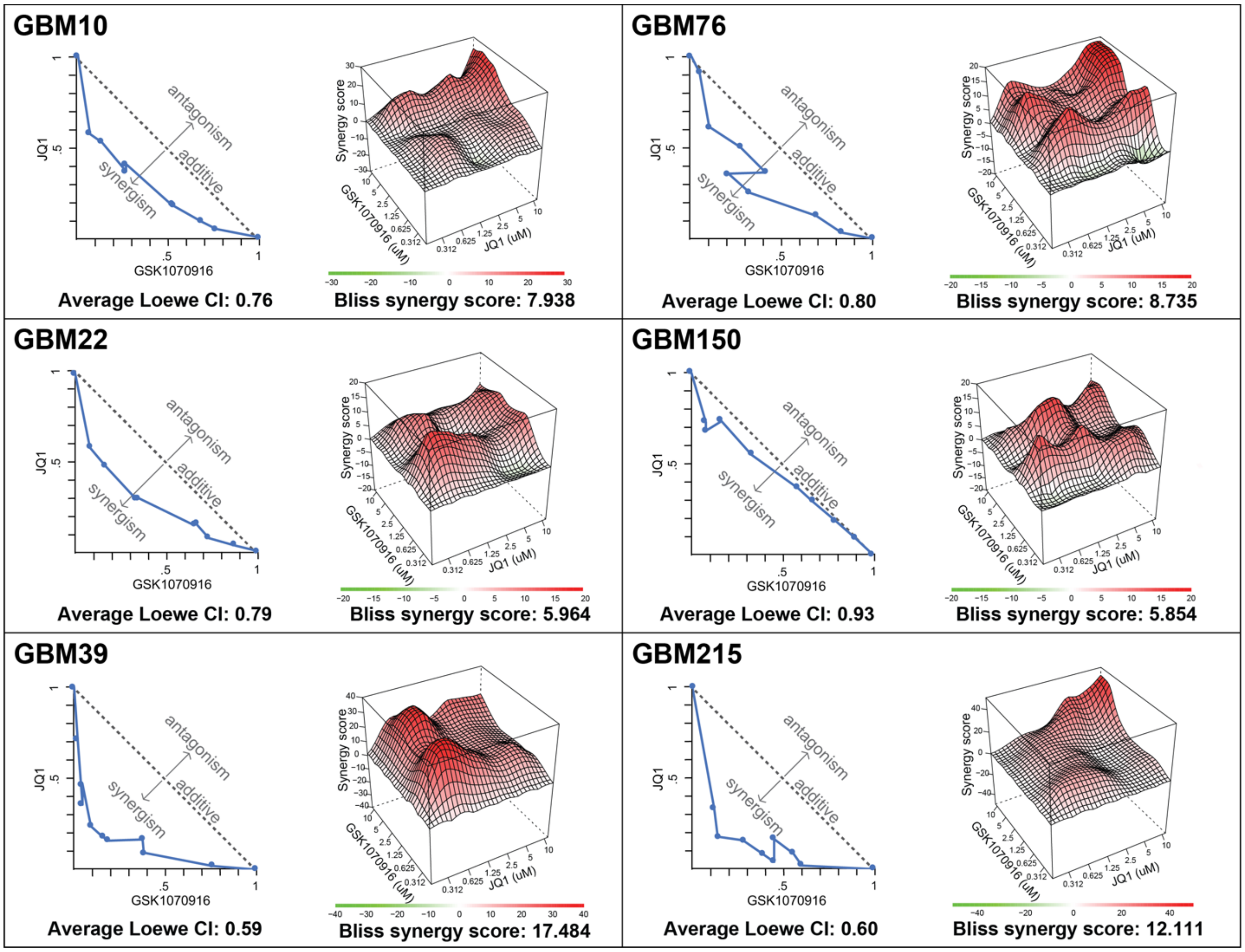

Supplementary Figure 4. Loewe and Bliss synergy plots of JQ1 and GSK1070916 combination matrices. GBM PDX cell lines were treated with JQ1 and/or GSK-1070916 using a combination matrix format with concentrations ranging from 10μM – 0μM for each compound. ATP levels were measured using CellTiter-Glo in 6 GBM PDX cell lines that were treated with JQ1 and/or GSK1070916. Combination index (CI) points in the isobologram that are below the diagonal are considered synergistic and a combination index (CI) < 1 indicates synergy. A positive Bliss Score (red) indicates synergy under the Bliss model between the two compounds at the corresponding dose ratio of the matrix and an increased synergy score indicates increased synergy. Source data can be found in the Source Data file, Supplementary Data 6, under tab Supplementary Figure 4.

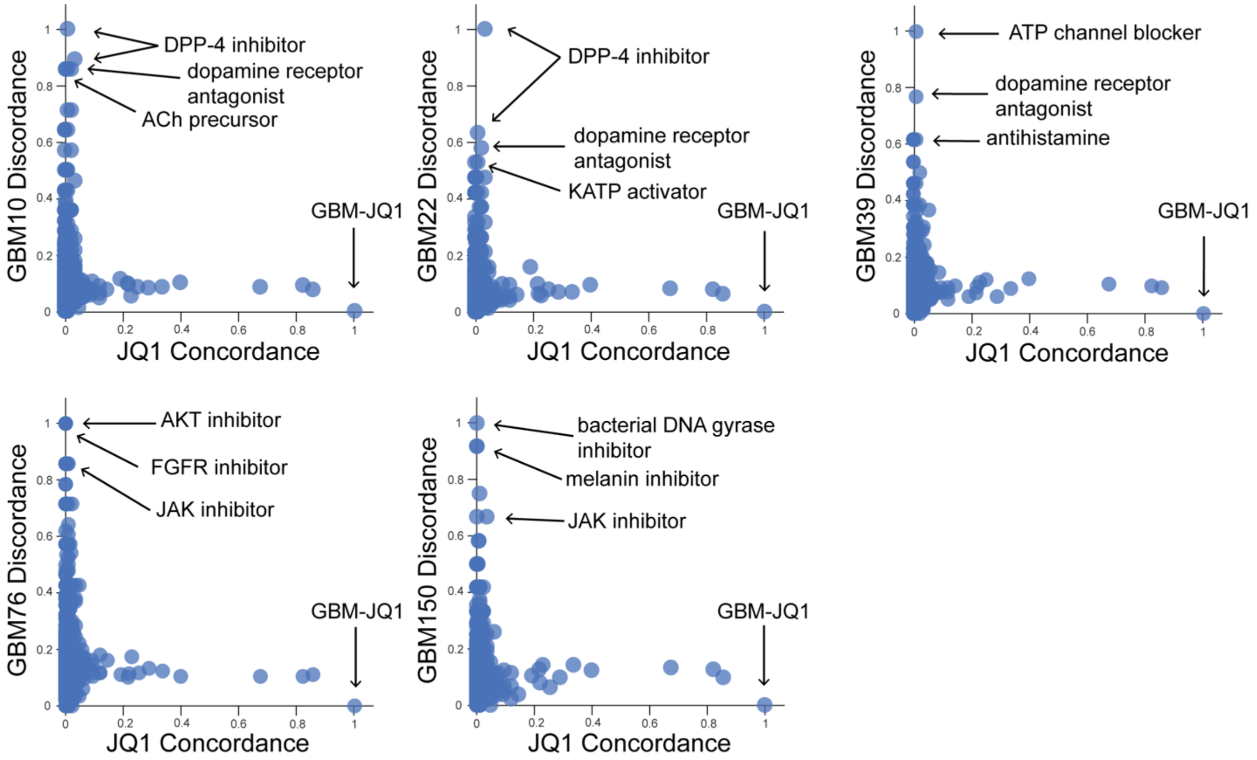

Supplementary Figure 5. Patient specific disease signatures identify compounds orthogonal to JQ1. The JQ1 label indicates the JQ1 signature that was calculated using the LINCS L1000 dataset. Compounds with a high x-axis value are similar to JQ1 signature and compounds with a high y-axis value are orthogonal to JQ1 signature. Top compounds are labeled based on mechanism of action. Source data can be found in the Source Data file, Supplementary Data 6, under tab Supplementary Figure 5.

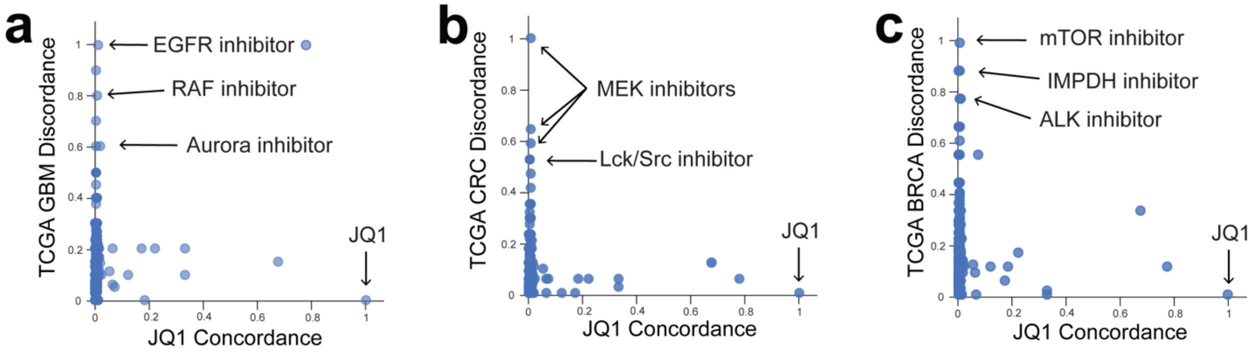

Supplementary Figure 6. Cancer type specific disease signatures identify compounds orthogonal to JQ1. The JQ1 label indicates the JQ1 signature that was calculated using the LINCS L1000 dataset. Compounds with a high x-axis value have a signature similar to JQ1, and compounds with a high y-axis value have a signature orthogonal to the disease signature. Plots were generated for the disease signature of **a.** glioblastoma (GBM), **b.** colorectal cancer (CRC), and **c.** breast cancer (BRCA). Source data can be found in the Source Data file, Supplementary Data 6, under tab Supplementary Figure 6.

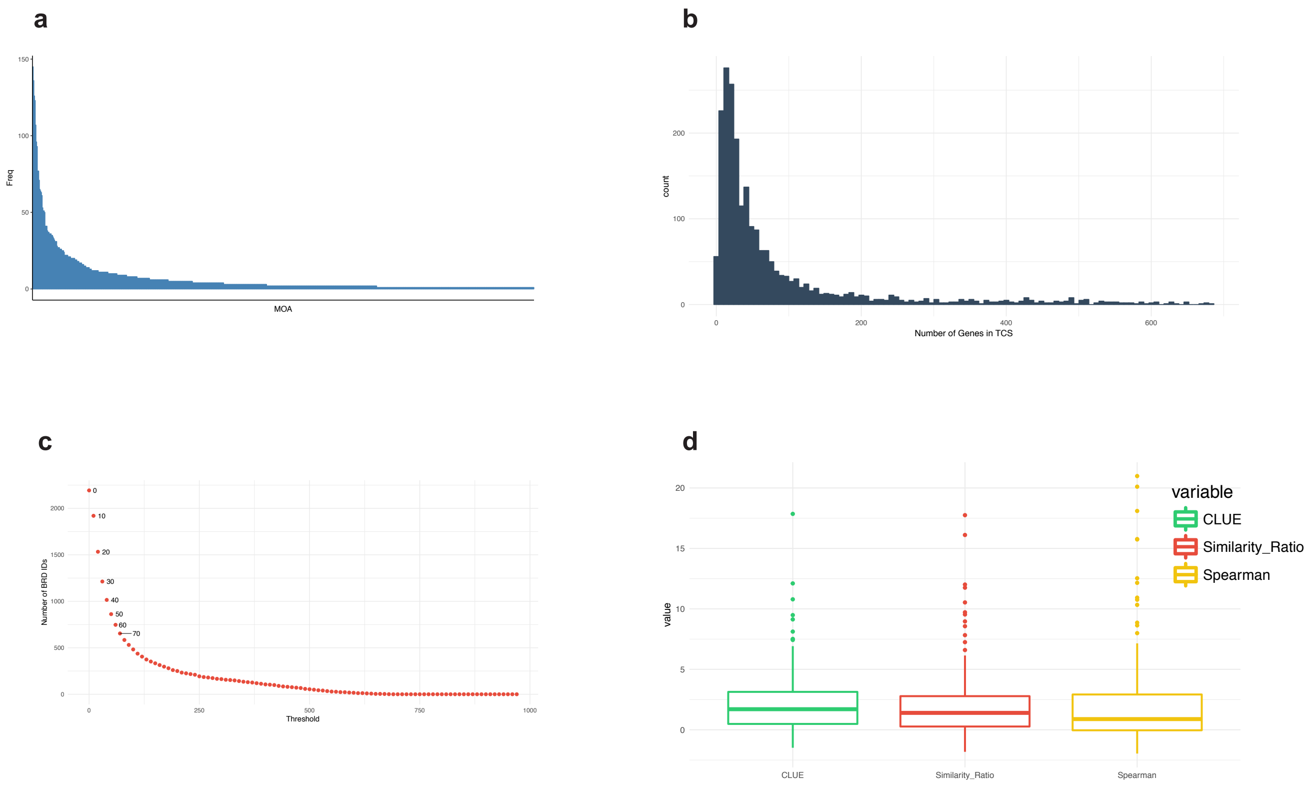

Supplementary Figure 7. Comparison of similarity between compounds of the same MOA, using different metrics. a. Histogram of number of small molecules that correspond to each MOA category. b. Histogram of the number of genes per TCS in the Phase 1 L1000 Dataset. c. The number of small molecules that pass a given gene TCS threshold. d. Difference (measured in z-scores) between observed and random similarity of small molecules belonging to the same MOA class using 3 similarity measures. CLUE uses the Broad Connectivity Score; Similarity Ratio and Spearman Correlation use the TCS of the same compounds.

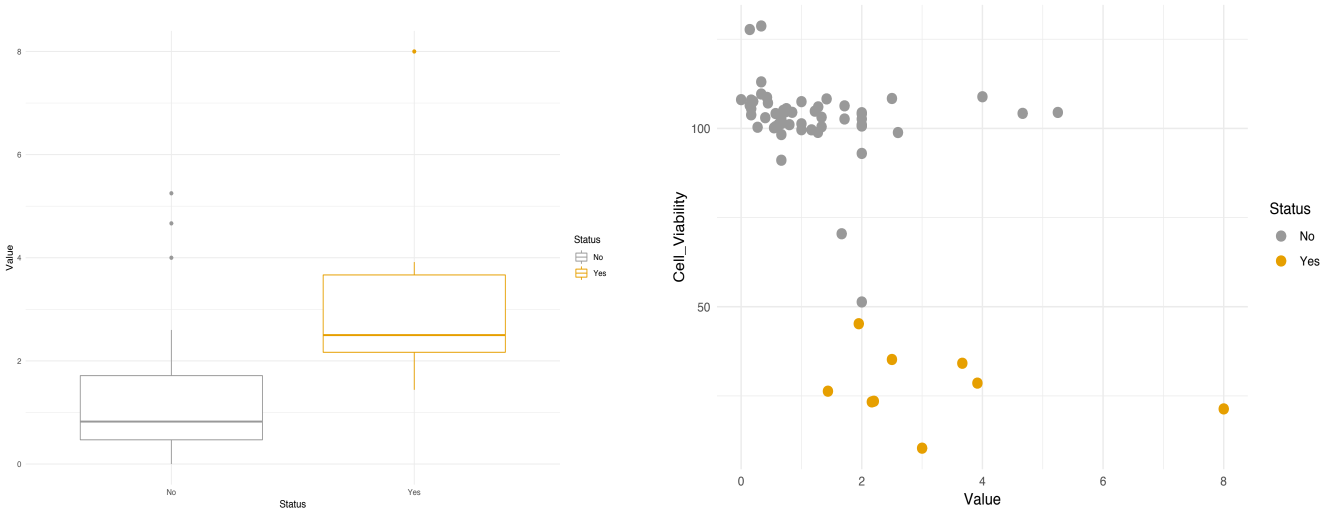

Supplementary Figure 8. Individual TCSs can predict sensitivity in glioblastoma cells. Left. Distribution of Individual Discordance Ratios between active (Yes, colored in grey) and non-active (No, colored in orange) compounds as labeled in the GBM dataset. Right. Cell Viability compared to Discordance Ratio for single-drug treatments in glioblastoma cells.

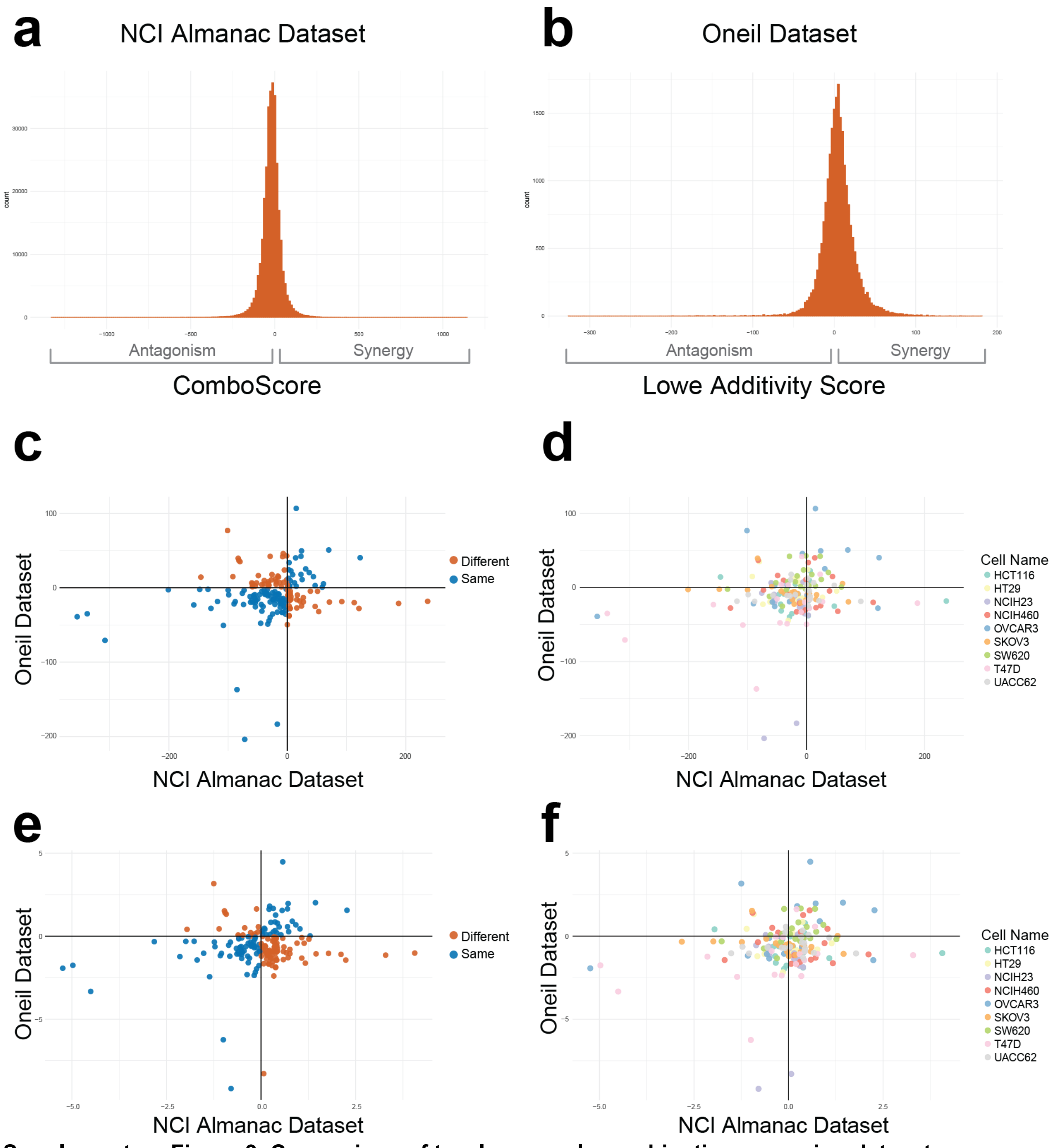

Supplementary Figure 9. Comparison of two large-scale combination screening datasets. a. Distribution of the ComboScore values for all combinations tested in the NCI ALMANAC dataset. **b.** Distribution of the Lowe Additivity Scores for all the combinations tested in the Oneil dataset. **c.** Plotting of the synergy score values for similar combinations (same drug1, drug2 & cell line) between the two datasets. Combinations that exhibited a positive synergy score in both datasets are colored in blue. x-axis: ComboScore from NCI ALMANAC, y-axis: Lowe Additivity Score from the Oneil Dataset **d.** Same as c but combinations are colored based on the cell line that was used. **e.** Plotting of the z-scores of the synergy scores for similar combinations between the two datasets. Combinations that exhibited a positive synergy score in both datasets are colored in blue. x-axis: ComboScore from NCI ALMANAC, y-axis: Lowe Additivity Score from the Oneil Dataset. **f.** Same as (e) but combinations are colored based on the cell line that was used.

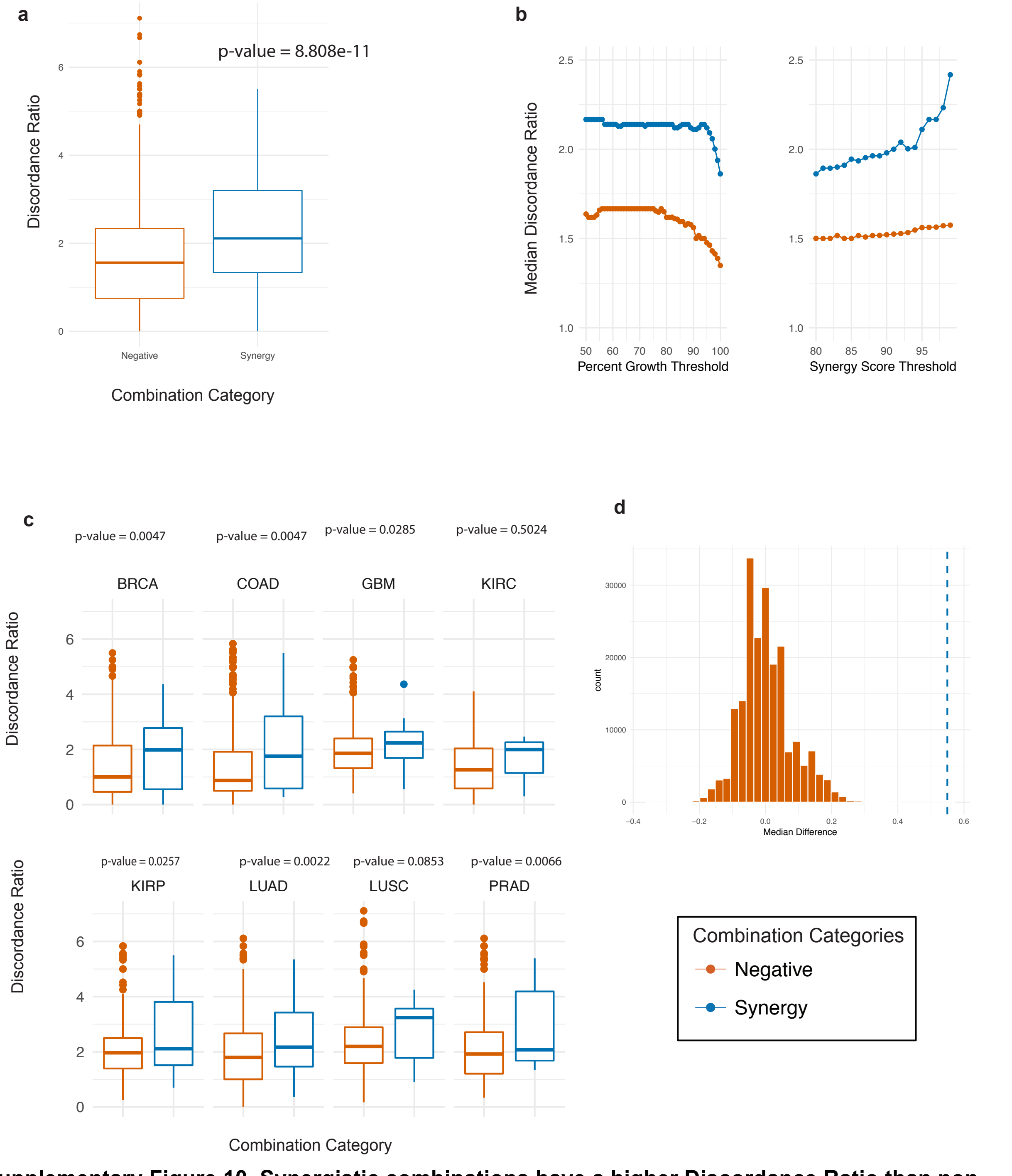

Supplementary Figure 10. Synergistic combinations have a higher Discordance Ratio than non-synergistic combinations. a. The NCI ALMANAC combinations were split into two categories (Synergy: top 5% of ComboScores, Negative: ComboScores below threshold) and then the Discordance Ratio distribution for each category was plotted. **b.** The Discordance Ratio medians of the Synergy and Negative categories, using different values of Synergy Score Threshold and Drug Activity filters **c.** Same as a, but divided according to the cancer type of each cell line used in the drug combinations. **d.** Histogram of the distribution of the difference in medians between the two categories after 200,000 permutations of the synergy and negative labels. The dotted line indicates the difference in the medians of the correct labeling. Two-sided Mann– Whitney–Wilcoxon test was used to assess the difference in Discordance Ratios between the two categories.

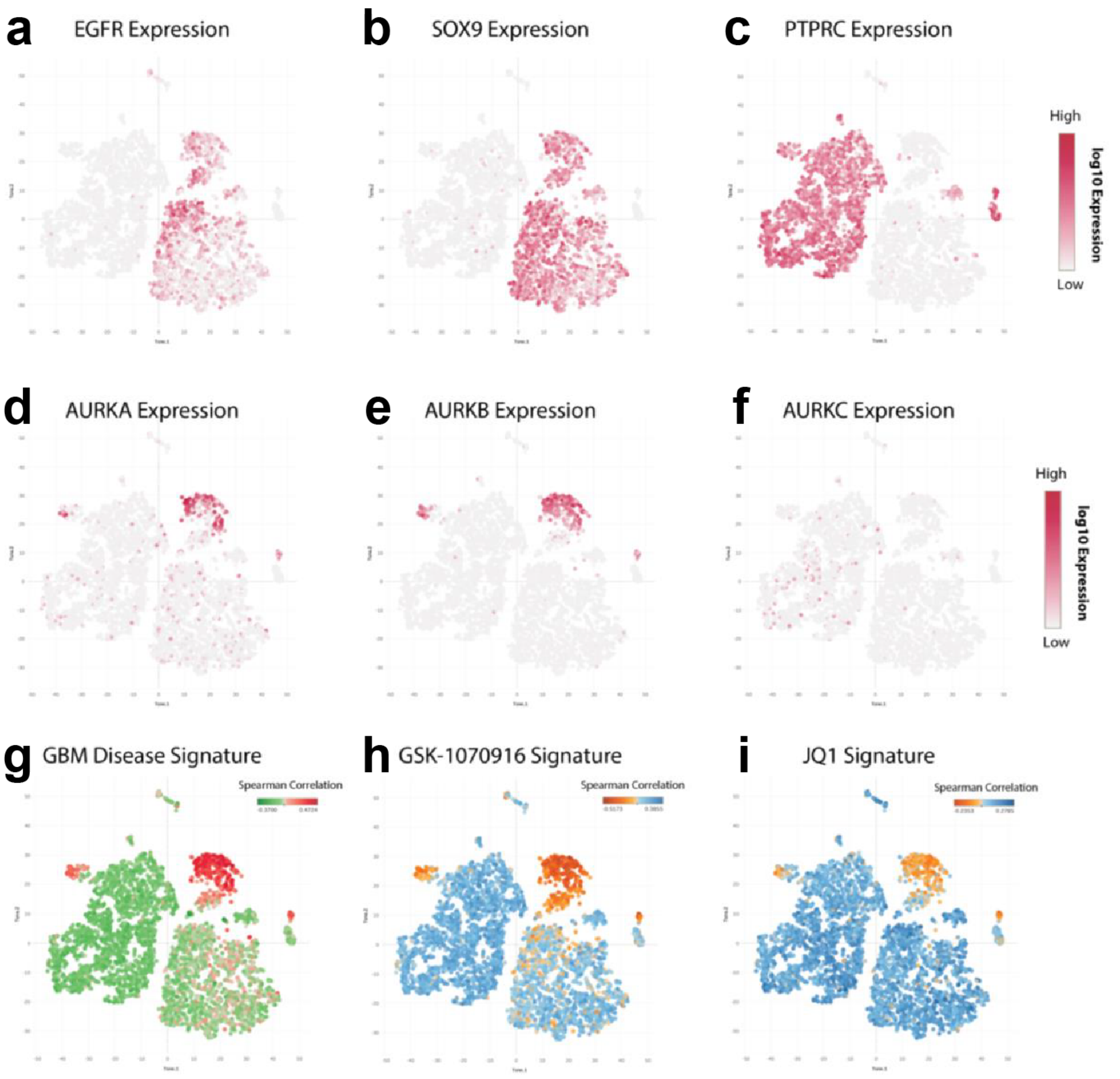

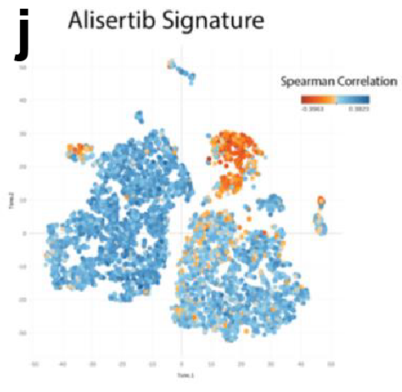

Supplementary Figure 11. Disease and drug signatures overlap neoplastic sub-populations from single-cell RNA-seq of a GBM sample. **a-c.** Expression of neoplastic-specific (EGFR, SOX9) and myeloid-specific (PTPRC) gene markers overlaid on the tSNE projection of 5966 Single cells of a GBM sample. **d-f.** Expression of Aurora Kinases (AURKA, AURKB, and AURKC respectively) on the same tSNE projection **g**. The Spearman Correlation of each single cell to the GBM Disease Signature, overlaid on the same tSNE plot. **h.** The Spearman Correlation of each single cell to the GSK-1070916 TCS, overlaid on the same tSNE plot. **i**. The Spearman Correlation of each single cell to the JQ1 TCS, overlaid on the same tSNE plot. **j.** The Spearman Correlation of each single cell to the Alisertib TCS, overlaid on the same tSNE plot. A GBM tumor was dissociated with papain and analyzed using the 10X Chromium System. Raw base call (BCL) files were analyzed using CellRanger (version 2.1.1). The *mkfastq* command was used to generate FASTQ files and the *count* command was used to generate gene expression matrices and CLOUPE visualization files aligned to the GRCh38 genome. Data was visualized by t-distributed stochastic neighbor embedding (tSNE) using the

Loupe Cell Browser output. Note that Aurora kinase A and Aurora kinase B are in the same cluster in the neoplastic part of the tumor but Aurora kinase C is sparsely expressed in the tumor.

SUPPLEMENTARY TABLES

	TMZ + Radiation	TMZ	Radiation	JQ1			SR1277				
**Upregulated**	PRR7	CCNE2	GTPBP8	CENPE	MYBL2	ST3GAL5	RAB21	LYN	CSK	KIAA1279	ETV1
	UBE2L6	CCNF	DERA	EPB41L2	SQSTM1	SPAG7	NR2F6	GADD45A	CCDC90A	ATP6V0B	ECD
	NFKBIE		SPDEF	SMAD3	ARHGEF12	SFN	NFE2L2	EPB41L2	FZD7	FBXO11	COASY
	FGFR4		STAMBP	SLC27A3	SCP2	ACOT9	H2AFV	GDPD5	GABPB1	ADI1	XPO7
	CASP2		PSMB8	PIN1	BAMBI	GABPB1	ILK	PLP2	TP53BP2	NCK2	USP1
	BIRC2		CEP57	DAXX	EDEM1	RFC5	MLEC	PTPRF	EIF4G1	PTPLAD1	HIST2H2BE
				NIT1	SLC35F2	FBXO7	KLHL21	GNAI1	SUPV3L1	RTN2	GPC1
				IKBKE	TEX10	XPO7	RFNG	UBE2C	G3BP1	FOXO4	GADD45B
				DDX10	MMP2	LPAR2	STAT3	YTHDF1	MALT1	SKP1	
				TOMM70A	RAE1		PRKACA	PAPD7	AXIN1	DHRS7	
				MSRA	IGHMBP2		HOXA5	MSH6	DCK	USP7	
				SLC35A3	HSD17B10		TIMP2	FOS	HLA.DMA	TBX2	
				NFKBIE	KDM5B		SORBS3	CREG1	CYTH1	ARID5B	
				CD97	PKIG		TRIB1	TERT	PTPN1	ISOC1	
				RRS1	RFX5		PRKAG2	TM9SF2	DUSP22	IER3	
**Downregulated**	HMGCR	PSMD9	PSMD9	DRAP1	DCK	DNAJC15	MYCBP2	EPRS	PCM1	WDR61	PPP2R3C
	ISOC1		CXCL2	ELAVL1	KIAA1033	TBPL1	CPNE3	FIS1	KDM5A	TOPBP1	BECN1
	BNIP3L		YTHDF1	PHGDH	USP6NL	NT5DC2	NUP88	TIMM17B	DHX29	TIMM9	RFC5
	CDC42		ZNF451	MBOAT7	SYNGR3	SUZ12	SMC4	GLOD4	MAPK9	HN1L	SKIV2L
	ALAS1		DHDDS	IGFBP3	LSM5	MYC	RB1	ATP6V1D	CDK2	PYGL	
			PPARG	PAF1	FOS	WASF3	KIF14	UBE3C	SCAND1	ARHGEF12	
			MRPS16	UBE3C	CHEK1	TMEM110	STUB1	PSME2	CCNH	KIAA0528	
			TFAP2A	PRKAG2	ICAM1	DERA	ST6GALNAC2	TP53BP1	RPA3	EIF5	
			WDR67	CSNK1A1	PRSS23	SLC25A14	RUVBL1	KEAP1	WDR67	ZNF451	
			DNM1L	SKIV2L	MRPS16	LOXL1	CPSF4	CCNA2	MCM3	PIN1	
			TEX10	KIF5C	SMC4	CDK4	LRRC16A	PHKB	CHMP6	MRPL12	
				IKBKAP	DHX29	EXOSC4	CEBPZ	CLTC	TGFBR2	PSMG1	
				ATMIN	CCNH	CDK19	TMEM5	NUSAP1	AKR7A2	ELOVL6	
				INTS3	COG2	PSME2	POP4	CHMP4A	SENP6	ELAC2	
				POLR2K	PACSIN3		ASCC3	PCMT1	PGM1	TOP2A	

Supplementary Table 1. Upregulated and downregulated L1000 genes in the GBM drug signatures.

SUPPLEMENTARY REFERENCES

1 Subramanian, A. *et al. A Next Generation Connectivity Map: L1000 Platform And The First 1,000,000 Profiles* (2017).

2 Corsello, S. M. *et al.* The Drug Repurposing Hub: a next-generation drug library and information resource. *Nat Med* **23**, 405-408, doi:10.1038/nm.4306 (2017).

3 Seashore-Ludlow, B. *et al.* Harnessing Connectivity in a Large-Scale Small-Molecule Sensitivity Dataset. *Cancer Discov* **5**, 1210-1223, doi:10.1158/2159-8290.CD-15-0235 (2015).

4 Gaulton, A. *et al.* The ChEMBL database in 2017. *Nucleic acids research* **45**, D945-D954, doi:10.1093/nar/gkw1074 (2017).

5 Jiang, P. *et al.* Novel anti-glioblastoma agents and therapeutic combinations identified from a collection of FDA approved drugs. *Journal of translational medicine* **12**, 13, doi:10.1186/1479-5876-12-13 (2014).

6 Schmidt, L. *et al.* Comparative drug pair screening across multiple glioblastoma cell lines reveals novel drug-drug interactions. *Neuro-oncology* **15**, 1469-1478, doi:10.1093/neuonc/not111 (2013).

7 Holbeck, S. L. *et al.* The National Cancer Institute ALMANAC: A Comprehensive Screening Resource for the Detection of Anticancer Drug Pairs with Enhanced Therapeutic Activity. *Cancer research* **77**, 3564-3576, doi:10.1158/0008-5472.CAN-17-0489 (2017).

8 O'Neil, J. *et al.* An Unbiased Oncology Compound Screen to Identify Novel Combination Strategies. *Mol Cancer Ther* **15**, 1155-1162, doi:10.1158/1535-7163.MCT-15-0843 (2016).

9 Preuer, K. *et al.* DeepSynergy: predicting anti-cancer drug synergy with Deep Learning. *Bioinformatics* **34**, 1538-1546, doi:10.1093/bioinformatics/btx806 (2018).

10 Mpindi, J. P. *et al.* Consistency in drug response profiling. *Nature* **540**, E5-E6, doi:10.1038/nature20171 (2016).

11 Haverty, P. M. *et al.* Reproducible pharmacogenomic profiling of cancer cell line panels. *Nature* **533**, 333-337, doi:10.1038/nature17987 (2016).

12 Haibe-Kains, B. *et al.* Inconsistency in large pharmacogenomic studies. *Nature* **504**, 389-393, doi:10.1038/nature12831 (2013).

13 Safikhani, Z. *et al.* Safikhani et al. reply. *Nature* **540**, E11-E12, doi:10.1038/nature20581 (2016).

14 Bouhaddou, M. *et al.* Drug response consistency in CCLE and CGP. *Nature* **540**, E9-E10, doi:10.1038/nature20580 (2016).

15 Weinstein, J. N. & Lorenzi, P. L. Cancer: Discrepancies in drug sensitivity. *Nature* **504**, 381-383, doi:10.1038/nature12839 (2013).

16 Wilkerson, M. D. & Hayes, D. N. ConsensusClusterPlus: a class discovery tool with confidence assessments and item tracking. *Bioinformatics* **26**, 1572-1573, doi:10.1093/bioinformatics/btq170 (2010).
