# Supplementary material for: Drug and disease signature integration identifies synergistic combinations in glioblastoma
Source: Nat Commun. 2018 Dec 14;9:5315. doi: 10.1038/s41467-018-07659-z (PMC6294341; doi:10.1038/s41467-018-07659-z)
Supplement: Supplementary file 3 — Description of Additional Supplementary Files [file 41467_2018_7659_MOESM3_ESM.docx]

**Description of Additional Supplementary Files**

**File Name:** Supplementary Data 1

**Description:** Normalized results of drug synergy screens and FDA-approved drug screens.

**File Name**: Supplementary Data 2

**Description:** SynergySeq output for PDX GBM76 discordance and Gemcitabine concordance.

**File Name**: Supplementary Data 3

**Description:** Comparison of TCSs with CLUE Connectivity Scores based on mechanism of action.

**File Name:** Supplementary Data 4

**Description:** L1000 compounds with their respective cluster number and mechanism of action.

**File Name:** Supplementary Data 5

**Description:** Complete list of Concordance Ratios for each Aurora kinase.

**File Name:** Supplementary Data 6

**Description:** Source data file
