# Supplementary material for: Drug and disease signature integration identifies synergistic combinations in glioblastoma
Source: Nat Commun. 2018 Dec 14;9:5315. doi: 10.1038/s41467-018-07659-z (PMC6294341; doi:10.1038/s41467-018-07659-z)
Supplement: Supplementary file 10 — Reporting Summary [file 41467_2018_7659_MOESM10_ESM.pdf]

nature research

Corresponding author(s): *Double-blind peer review submissions: write DBPR and your manuscript number here instead of author names.*

Reporting Summary

Nature Research wishes to improve the reproducibility of the work that we publish. This form provides structure for consistency and transparency in reporting. For further information on Nature Research policies, see Authors & Referees and the Editorial Policy Checklist.

Statistical parameters

When statistical analyses are reported, confirm that the following items are present in the relevant location (e.g. figure legend, table legend, main text, or Methods section).

n/a	Confirmed	
☐	☒	The exact sample size (*n*) for each experimental group/condition, given as a discrete number and unit of measurement
☐	☒	An indication of whether measurements were taken from distinct samples or whether the same sample was measured repeatedly
☐	☒	The statistical test(s) used AND whether they are one- or two-sided<br>*Only common tests should be described solely by name; describe more complex techniques in the Methods section.*
☒	☐	A description of all covariates tested
☐	☒	A description of any assumptions or corrections, such as tests of normality and adjustment for multiple comparisons
☒	☐	A full description of the statistics including central tendency (e.g. means) or other basic estimates (e.g. regression coefficient) AND variation (e.g. standard deviation) or associated estimates of uncertainty (e.g. confidence intervals)
☐	☒	For null hypothesis testing, the test statistic (e.g. *F*, *t*, *r*) with confidence intervals, effect sizes, degrees of freedom and *P* value noted<br>*Give P values as exact values whenever suitable.*
☒	☐	For Bayesian analysis, information on the choice of priors and Markov chain Monte Carlo settings
☒	☐	For hierarchical and complex designs, identification of the appropriate level for tests and full reporting of outcomes
☐	☒	Estimates of effect sizes (e.g. Cohen's *d*, Pearson's *r*), indicating how they were calculated
☐	☒	Clearly defined error bars<br>*State explicitly what error bars represent (e.g. SD, SE, CI)*

Our web collection on statistics for biologists may be useful.

Software and code

Policy information about availability of computer code

Data collection	Data sources are described in Material and Methods
Data analysis	Algorithms and Software used are described in Material and Methods

For manuscripts utilizing custom algorithms or software that are central to the research but not yet described in published literature, software must be made available to editors/reviewers upon request. We strongly encourage code deposition in a community repository (e.g. GitHub). See the Nature Research guidelines for submitting code & software for further information.

Data

Policy information about availability of data

All manuscripts must include a data availability statement. This statement should provide the following information, where applicable:

- Accession codes, unique identifiers, or web links for publicly available datasets
- A list of figures that have associated raw data
- A description of any restrictions on data availability

The data that support the findings of this study are included in the Source Data File, the Suppl. Files 1 &2 and in public repositories with accession numbers listed in Material and Methods section.

Field-specific reporting

Please select the best fit for your research. If you are not sure, read the appropriate sections before making your selection.

☒ Life sciences ☐ Behavioural & social sciences ☐ Ecological, evolutionary & environmental sciences

For a reference copy of the document with all sections, see nature.com/authors/policies/ReportingSummary-flat.pdf

Life sciences study design

All studies must disclose on these points even when the disclosure is negative.

Sample size	In vivo: ten samples per group were used.<br>In vitro: four replicates per condition were used.
Data exclusions	In vivo: Animals were excluded if the tumor size exceeded 300mm2 at day 10 post implantation, if tumor size exceeded 2000mm2 at day 23, or if animal expired before tumor measurements were obtained at 23 days.<br>In vitro: outliers were excluded if standard deviation exceeded 10.
Replication	In vivo: measurements were verified by examining tumor size 2x and weighing mice 2x.<br>In vitro: at least 2 replicates with a standard deviation less than 10 were used per data point.
Randomization	Mice were binned into groups based on 10 day starting tumor size, then randomly distributed into treatment groups with an equal distribution of males and females.
Blinding	Investigators were blinded to the treatment groups for all in vivo measurements. Blinding was not important for in vitro studies because the output was raw luminescent values.

Reporting for specific materials, systems and methods

Materials & experimental systems

n/a	Involved in the study	
☒	☐	Unique biological materials
☒	☐	Antibodies
☐	☒	Eukaryotic cell lines
☒	☐	Palaeontology
☐	☒	Animals and other organisms
☒	☐	Human research participants

Methods

n/a	Involved in the study	
☒	☐	ChIP-seq
☒	☐	Flow cytometry
☒	☐	MRI-based neuroimaging

Eukaryotic cell lines

Policy information about cell lines

Cell line source(s)	PDX National Resource at the Mayo Clinic
Authentication	Cell lines are authenticated by the PDX National Resource on a bi-annual basis. Short-tandem repeat (STR) was used to compare the original patient tumor sample, when available, with the derivative PDX model to ensure the provenance of the PDX line. STR analysis from early passage PDX tissue was used to establish a gold standard identity for each PDX line, and these data are publicly available.
Mycoplasma contamination	The cell lines were not tested for mycoplasma contamination.
Commonly misidentified lines<br>(See ICLAC register)	n/a

Animals and other organisms

Policy information about studies involving animals; ARRIVE guidelines recommended for reporting animal research

Laboratory animals	Crl:NU-Foxn1nu mice from Charles River Laboratory, 50% males, 50% females

Wild animals	n/a
Field-collected samples	n/a
